# To investigate the effect of neck-shaft angle in surgical hip dislocation combined with femoral neck rotational osteotomy in the treatment of osteonecrosis of the femoral head and to combine with finite element analysis

**DOI:** 10.3389/fbioe.2025.1495292

**Published:** 2025-03-19

**Authors:** Sun Jiahao, Ma Bowen, Zhang Chiyu, Xia Tianwei, Shen Jirong, Zhang Chao

**Affiliations:** Affiliated Hospital of Nanjing University of Traditional Chinese Medicine, Nanjing, Jiangsu, China

**Keywords:** osteonecrosis of the femoral head, surgical hip dislocation, femoral neck rotational osteotomy, neck-shaft angle, efficacy of hip preservation

## Abstract

**Background:**

The Neck-shaft Angle (NSA) is a reliable predictor of the outcome of internal fixation for femoral neck fractures. Surgical Hip Dislocation Combined with Femoral Neck Rotational Osteotomy (SHD-FNRO) is an effective surgical method for treating femoral head necrosis. However, the potential role of NSA in predicting the outcomes of hip preservation after SHD-FNRO has not been explored.

**Objective:**

This study aims to investigate the value of NSA in predicting the outcomes of hip preservation after SHD-FNRO by comparing the results among different patients with osteonecrosis of the femoral head (ONFH) who were treated with SHD-FNRO.

**Methods:**

We retrospectively analyzed clinical data from 33 patients (33 hips) who underwent SHD-FNRO at our hospital between January 2017 and December 2021. Based on the outcome of hip preservation, patients were divided into two groups: group A (successful hip preservation) and group B (failed hip preservation). Statistical analysis was performed to evaluate any differences between these groups. Additionally, three-dimensional models with varying NSA values were created to analyze changes in maximum displacement and stress on the osteotomy surface.

**Results:**

During a mean follow-up period of approximately 40 months, successful hip preservation was achieved in 26 patients while 7 patients experienced failure. There was a statistically significant difference in NSA between these two groups (P < 0.05). Biomechanical analysis demonstrated a close relationship between NSA and postoperative biomechanical changes.

**Conclusion:**

The size of NSA is closely associated with the success rate of hip preservation when treating osteonecrosis of the femoral head using SHD-FNRO. Therefore, careful attention should be given to selecting an appropriate osteotomy surface that can adjust NSA size effectively, thus achieving better outcomes for hip preservation.

## 1 Introduction

Osteonecrosis of the femoral head (ONFH) is a debilitating arthropathy with high morbidity, commonly affecting young and middle-aged populations. Without proper diagnosis and treatment, approximately 80% of ONFH patients will experience collapse, significantly impairing their quality of life and necessitating artificial total hip arthroplasty. However, due to limited prosthesis lifespan and increasing complications, revision becomes inevitable for young and middle-aged patients. Therefore, enhancing the success rate of hip preservation treatments remains a crucial focus in the clinical research on ONFH (Cardín-Pereda et al., 2022; Chau et al., 2021; Xue et al., 2019).

The neck-shaft Angle (NSA) plays a crucial role in the internal fixation of femoral neck fractures, as it not only affects the stability, healing process, and postoperative complications but also influences the recovery of hip joint function. Restoring the normal NSA range (110°–140° in adults with a mean of 127°) is considered essential for promoting anatomical alignment, accelerating healing, and reducing complications such as loosening of internal fixation, nonunion of fractures, and femoral head necrosis (Fang et al., 2023; Oh et al., 2017). Surgical Hip Dislocation Combined with Femoral Neck Rotational Osteotomy (SHD-FNRO), developed based on Sugioka’s improved intertrochanteric rotational osteotomy proposed in 1976 (Sugioka et al., 1992), involves truncating the femoral neck to relocate necrotic areas away from weight-bearing regions and fixing the osteotomy using internal fixation. However, whether NSA can predict outcomes in SHD-FNRO hip preservation remains unexplored.

Therefore, this retrospective study analyzed clinical data from 33 patients (33 hips) who underwent SHD-FNRO at our hospital between January 2017 and December 2021 to evaluate the impact of NSA on hip preservation outcomes and biomechanical changes under different NSA conditions.

## 2 Data and methods

### 2.1 Patient selection criteria

**FIGURE 1:**
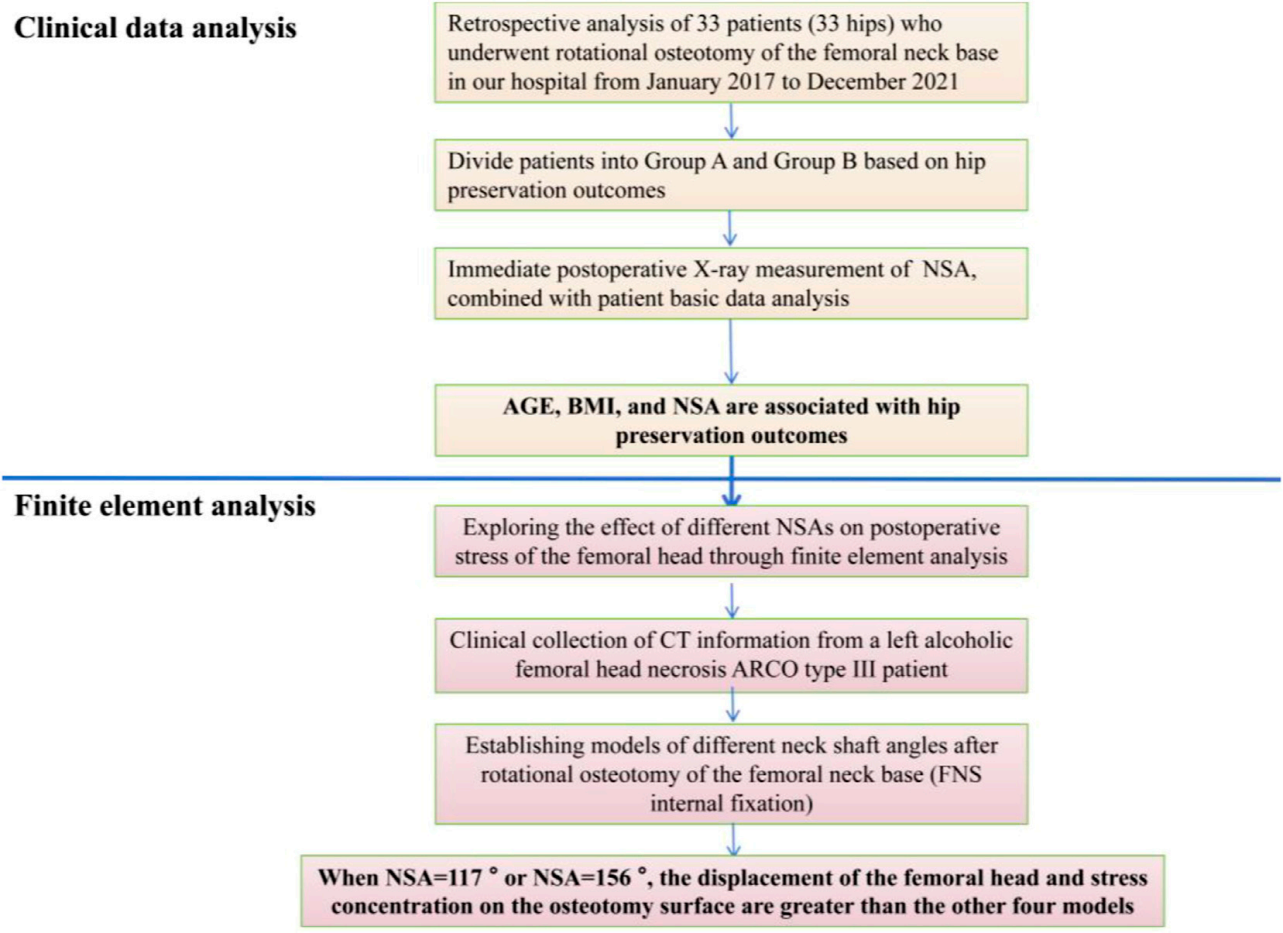
Research ideas.

The research roadmap is shown in Figure 1.

#### 2.1.1 Inclusion criteria

(1) Patients with femoral head necrosis at ARCO (Association Research Circulation Osseous) Stage III, with the necrotic area located in the anterolateral portion of the femoral head and without collapse.
(2) Patients demonstrating good blood supply to the femoral head on preoperative DSA or dynamic contrast-enhanced MRI.
(3) Patients whose postoperative DSA or dynamic contrast-enhanced MRI shows no significant impairment of femoral head blood supply due to the surgery.

#### 2.1.2 Exclusion criteria

(1) Patients who are unwilling or unable to comply with follow-up.
(2) Patients who received other treatments during the follow-up period.
(3) Patients with hip preservation failure within 6 months after surgery.
(4) Patients who experienced severe adverse reactions, allergies, or unexpected events.
(5) Patients with missing or incomplete imaging data.
  A total of 33 patients (33 hips) who underwent SHD-FNRO from 01/01/2017 to 01/12/2021 were included in this study.

### 2.2 Surgical methods

**FIGURE 2:**
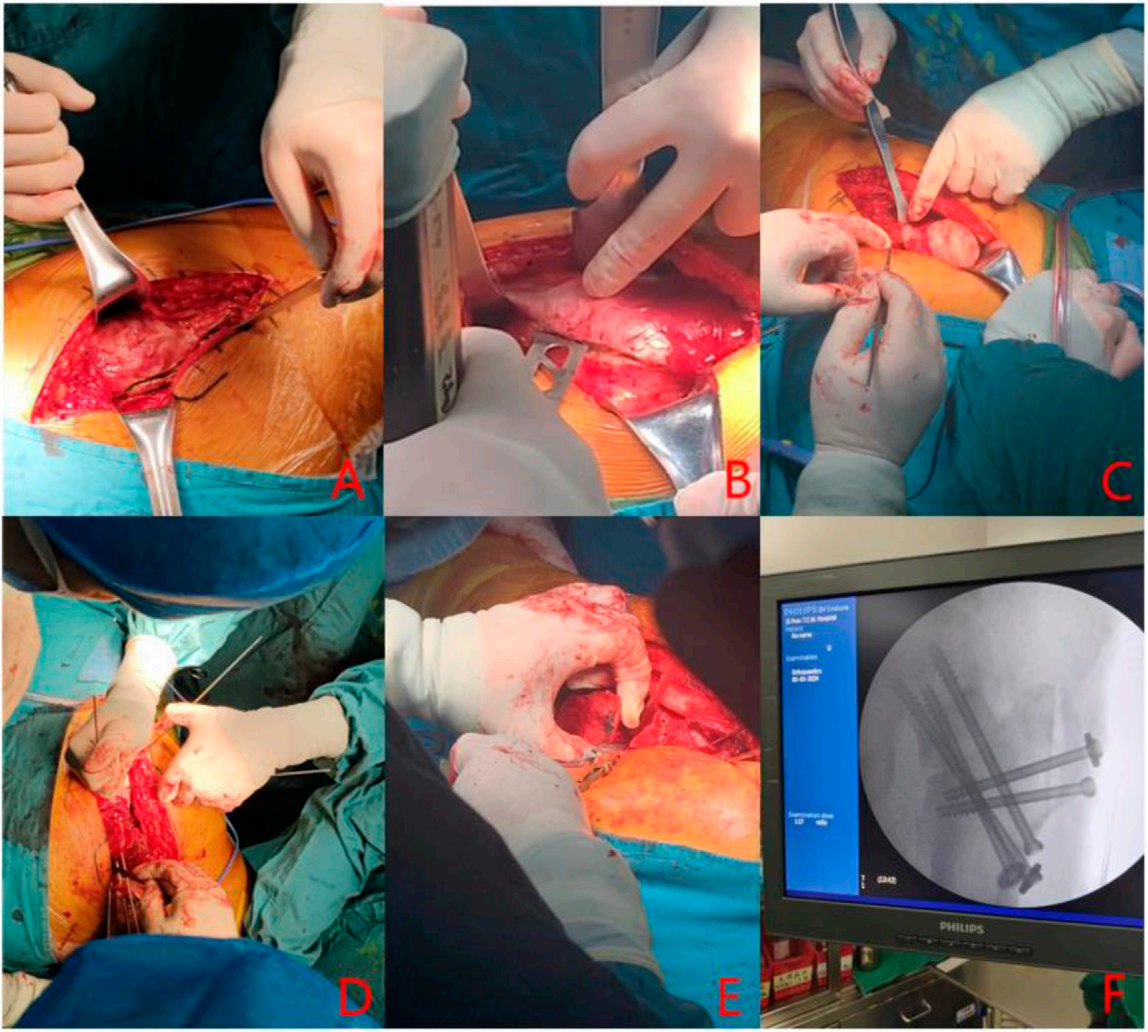
**(A)** lateral hip incision; **(B)** Osteotomy at the greater trochanter; **(C)** femoral head dislocation; **(D)** Kirschner needle drilling; **(E)** Osteotomy at the base of femoral neck; **(F)** Intraoperative fluoroscopy.

Following preoperative planning and under general anesthesia, the patient is positioned in a lateral decubitus position. A lateral incision is made to expose and protect the insertion sites of the gluteus medius and rectus femoris muscles. An osteotomy is performed at the greater trochanter, ensuring the preservation of both the proximal and distal insertions. The anterior femoral neck is then exposed, and the joint capsule is incised to inspect for acetabular labral damage. The femoral head is dislocated to visualize the anterolateral collapse. A Kirschner wire is used to probe the femoral head, assessing its blood supply. Soft tissue flaps from the posterior aspect of the greater trochanter are excised to fully expose the posterior osteotomy site at the base of the femoral neck. With the base of the femoral neck as the axis, the femoral head is rotated anteriorly or posteriorly under intraoperative fluoroscopic guidance to transfer the necrotic area beneath it, achieving a satisfactory lateral weight-bearing region. The femoral neck is securely fixed using either cannulated screws or a Dynamic Hip Screw (DHS) system, or the Femoral Neck System (FNS). The joint capsule is sutured, and the greater trochanter osteotomy site is secured with screws. See Figure 2 for details.

### 2.3 Patient data collection

#### 2.3.1 Measurement of NSA

Two independent orthopedic physicians performed measurements of the NSA on anteroposterior hip radiographs immediately following surgery. The measurements were conducted using Picture Archiving and Communication System (PACS) equipped with an angular measurement tool. The procedure involved the following steps:Identification of Reference Lines: The longitudinal axis of the femoral shaft was determined by drawing a line through the center points of the proximal and distal regions of the femoral shaft. The axis of the femoral neck was established by drawing a line through the center points of the femoral head and the narrowest part of the femoral neck. Measurement of the NSA: Using the angular measurement tool within the software, the angle between the femoral shaft axis and the femoral neck axis was measured. To ensure accuracy, magnification corrections were applied when necessary. Both observers received standardized training on NSA measurements using this method, and their results were cross-checked and reviewed by a senior orthopedic physician to ensure consistency and reliability.

All AP radiographs were taken with the patient in a supine position, with both lower extremities in a neutral position (15° of internal rotation). This standardized positioning minimizes femoral rotation artifacts, ensuring consistency and reliability in NSA measurements. Additionally, while 3D CT reconstructions provide detailed anatomical information, they are associated with higher costs, increased radiation exposure, and limited availability in routine clinical practice compared to plain radiographs. Therefore, in order to increase the generalizability of the results, we selected the AP position radiographs immediately after surgery to measure NSA.

#### 2.3.2 Selection of covariates

Preoperative demographic data including age, gender, etiology, BMI, smoking history, drinking history, hormone use history, and postoperative hormone use were collected for covariate selection. Additionally, postoperative serum biochemical indicators along with SHD-FNRO internal fixation methods (three nails, two nails, FNS or DHS) and hip preservation time were recorded.

#### 2.3.3 Postoperative follow-up

The follow-up data (X-ray, CT, blood biochemical indexes, etc.) were collected. The interval of follow-up was determined according to clinical needs and patients’ conditions, usually at 1 month, 3 months, 6 months, 12 months after surgery and once a year thereafter. To ensure that patients did not use any other hip-preserving treatment between each follow-up visit, regular communication and reminders were provided by telephone, text, or mail during follow-up. To ensure that patients did not receive additional hip-preserving treatments, we studied them by reviewing their medical records.

### 2.4 Finite element analysis

#### 2.4.1 Establishment of SHD-FNRO model: take FNS as an example

Image source: The CT data of a patient diagnosed with ARCO III type of left alcoholic necrosis of the femoral head were collected. The patient was a 30-year-old male weighing 80 kg, who had a history of heavy alcohol consumption for 5 years. CT scanning parameters included a layer thickness of 1 mm and a scanning range from the upper pelvic margin to 15 cm below the lesser trochanter. The acquired CT data were saved in DICOM format, and the original data were duplicated.

Software: Mimics21.0 software, Geomagic Studio 2017 software, Solidworks 2021 software, ANSYS 17.0 software.

**FIGURE 3:**
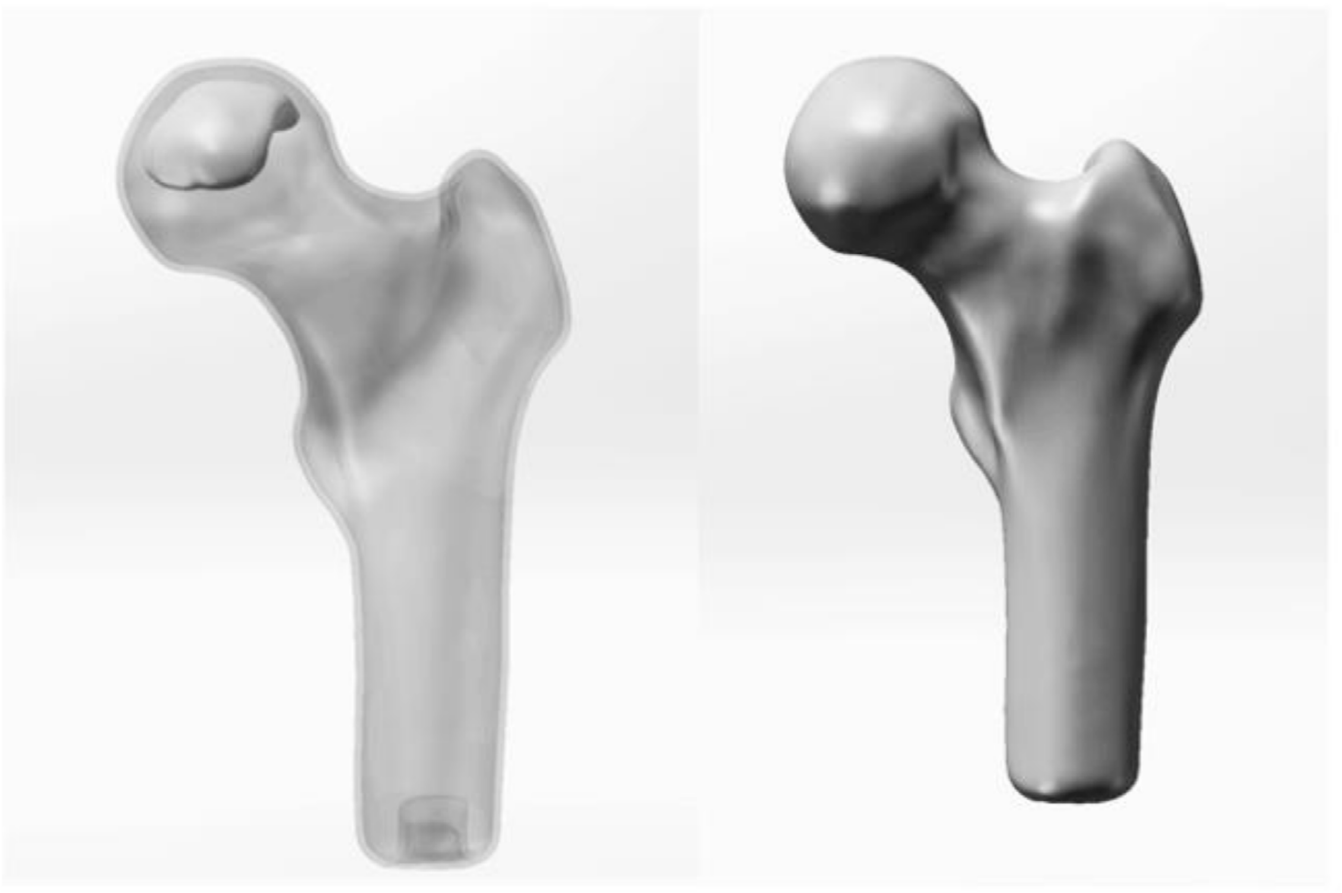
Preoperative geometric model.

Preoperative modeling: In Mimics Research 21.0, CT images were segmented using a specific threshold value to obtain an initial model of the femoral head. The initial model was then imported into Geomagic2017 to establish the preliminary geometric model, followed by noise reduction, removal of region features, and sanding processes to optimize the femoral head model. Subsequently, the optimized model was imported into SolidWorks 2021 for surface fitting and assembly. The final preoperative geometric model (Figure 3) was established and imported into ANASYS17.0 software for material assignment, body mesh formation, surface mesh generation, and subsequent finite element analysis (89,005 units and 89,005 nodes after mesh division).

**FIGURE 4:**
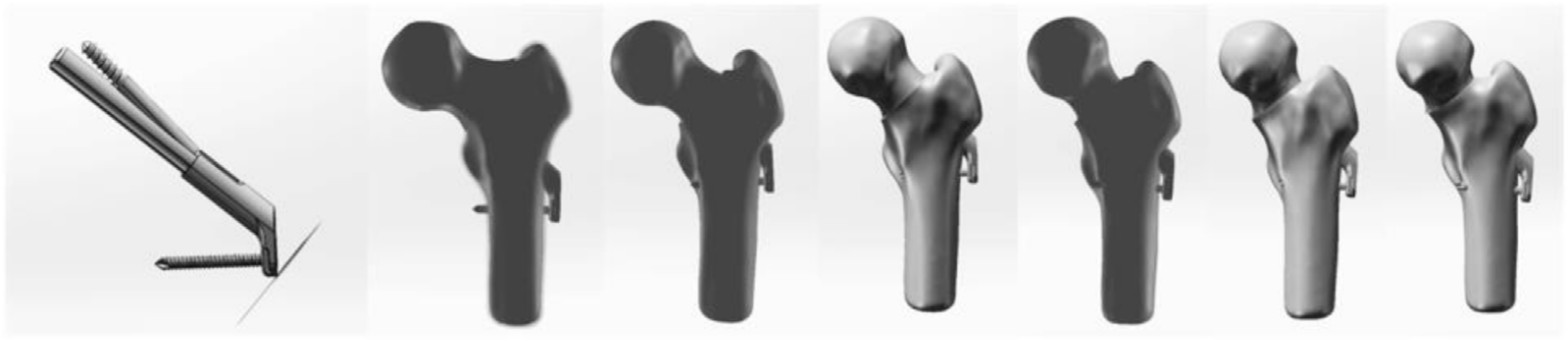
Postoperative models of FNS internal fixation and 6 kinds of SHD-FNRO (117°, 127°, 138°, 145°, 156°, 165°).

Establishment of different cervical trunk Angle models and FNS models after SHD-FNRO operation: The femur model was imported into the 3D engineering software SolidWorks 2021 as a STEP file. Following the SHD-FNRO procedure, a cross section was created at the base of the femoral neck, ensuring that the osteotomy surface was perpendicular to the axis of the femoral neck. Subsequently, by rotating the osteotomy block forward along the axis of the femoral neck, transfer of necrotic area out of weight-bearing region was achieved. The proximal and distal osteotomy surfaces were then closed together. Six different NSA models (117°, 127°, 138°, 145°, 156°, and 165°) were established based on varying angles of osteotomy surface. An internal fixation device model was developed using Johnson and Johnson’s FNS data as reference. This product consisted of a femoral neck bone plate, a femoral neck power rod, and a femoral neck anti-rotation screw. The FNS model was assembled with the femoral osteotomy model and placed within it according to clinical fixation methods to create the final model (Figure 4). Material assignment was performed in ANASYS17.0 software followed by volume meshing and surface meshing before conducting finite element analysis.

#### 2.4.2 Material property assignment

The material parameters for cortical bone, cancellous bone, necrotic zone, and internal fixation were inputted into ANSYS 17.0, with corresponding material properties assigned to each component of the analysis. The FNS internal fixation device was fabricated using titanium alloy material (Table 1) (Yadav et al., 2023).

**TABLE 1:** Material parameters.

Material name	Elastic modulus	Poisson’s ratio	References
Cortical bone	16,800	0.30	Song et al. (2023)
Cancellous bone	840	0.30	Song et al. (2023)
Necrosis	332.9	0.30	Song et al. (2023)
Titanium alloy	10,500	0.30	Song et al. (2023)

#### 2.4.3 Loading load and setting constraints

**FIGURE 5:**
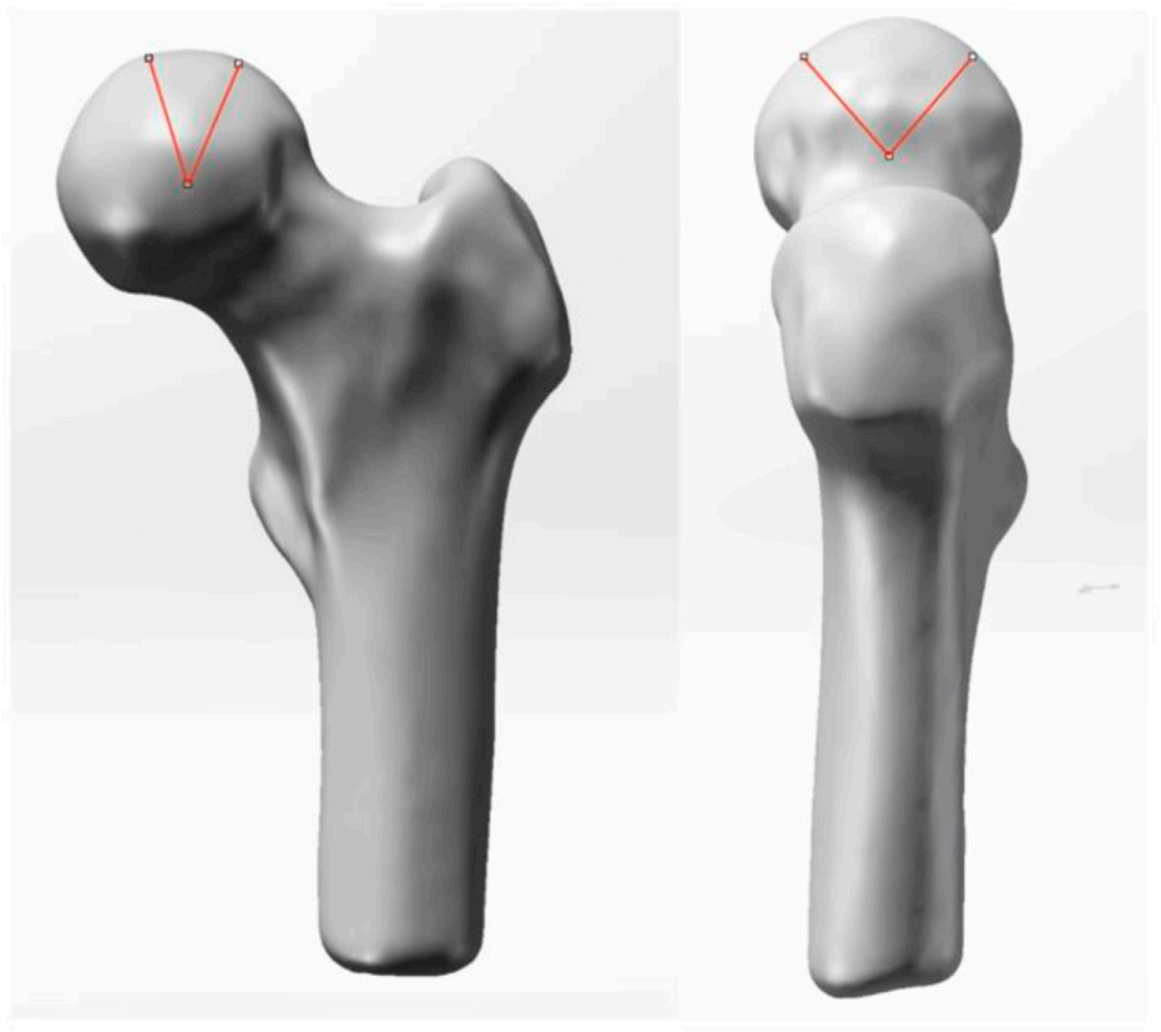
Areas of stress.

According to relevant biomechanical studies, the unilateral loading pressure is calculated as 2.5 times the product of body weight (M), gravity coefficient (G), and a constant value of 0.5N. In this study, loads of 920N, 1500N, and 2000N were applied to the weight-bearing area above the femoral head to investigate the biomechanical changes following rotational osteotomy under different stress conditions. The weight-bearing area was defined as an arc spanning 40° in both internal and external directions from the center of the femoral head, as well as an arc spanning 80° in the anterior-posterior direction relative to its center (Figure 5) (Brown et al., 1993). The distal end of the femur was immobilized as a fixed interface.

#### 2.4.4 Evaluation index

Examine the variations in maximum displacement values and stress concentration phenomena at the osteotomy surface across different models of SHD-FNRO.

### 2.5 Statistical methods

The statistical analysis of the data was performed using SPSS 27.0 software and GraphPad Prism 8. The normality of the distribution of continuous variables was assessed using the Schapiro-Wilke test. For variables with a symmetric distribution, mean (mean) and standard deviation (SD) were used as representations, while median and quartile ranges (IQR) were used for non-normal distributions. Disaggregated data were presented in absolute numbers and percentages. Statistical comparison of continuous variables involved Student’s t-test for normally distributed variables and Mann-Whitney U test for non-normally distributed variables. Univariate and multivariate logistic regression analyses were conducted to explore the relationship between exposure factors and outcome indicators.

## 3 Results

A retrospective analysis was conducted on 40 patients with femoral head necrosis treated with Surgical Hip Dislocation Combined with Femoral Neck Rotational Osteotomy (SHD-FNRO). Based on the inclusion and exclusion criteria, a total of 33 patients (33 hips) with femoral head necrosis were ultimately enrolled in the study. Among them, there were 26 male patients (26 hips) and 7 female patients (7 hips), aged between 18 and 48 years (average age, 31.8 years). The types of avascular necrosis of the femoral head (ANFH) were as follows: ischemic in 14 cases (14 hips), steroid-induced in 9 cases (9 hips), alcohol-induced in 7 cases (7 hips), and post-traumatic in 3 cases (3 hips). The methods of rotational osteotomy fixation were as follows: 19 patients received fixation with cannulated screws, 8 patients with Dynamic Hip Screw (DHS), and 6 patients with Femoral Neck System (FNS).

During follow-up, there were 7 cases of failed hip preservation, including 4 cases of further collapse of the femoral head, 1 case of screw loosening, and 2 cases of nonunion of the osteotomy site, which subsequently underwent Total Hip Arthroplasty (THA). According to the outcomes of hip preservation, the 33 patients were divided into two groups, and the specific details are shown in Table 2.

**TABLE 2:** Patient data.

Group	A	B	SD	P-value
N	26	7		
SEX			0.511 (−0.333, 1.354)	0.346
Male	23 (88.462%)	7 (100.000%)		
Female	3 (11.538%)	0 (0.000%)		
AGE	30.885 ± 5.949	35.286 ± 3.302	0.915 (0.052, 1.778)	0.034
Diagnose			0.289 (−0.548, 1.127)	0.925
Hormonal	7 (26.923%)	2 (28.571%)		
Alcoholic	6 (23.077%)	1 (14.286%)		
Traumatic	2 (7.692%)	1 (14.286%)		
Ischemic	11 (42.308%)	3 (42.857%)		
BMI	23.924 ± 2.499	27.831 ± 1.884	1.766 (0.829, 2.703)	<0.001
Drinking history			0.126 (−0.709, 0.961)	0.763
No	20 (76.923%)	5 (71.429%)		
Yes	6 (23.077%)	2 (28.571%)		
Smoking history			0.858 (−0.002, 1.718)	0.299
No	19 (73.077%)	7 (100.000%)		
Yes	7 (26.923%)	0 (0.000%)		
Hormone use history			0.339 (−0.500, 1.178)	0.646
No	19 (73.077%)	4 (57.143%)		
Yes	7 (26.923%)	3 (42.857%)		
Whether to continue taking hormones after surgery			0.634 (−0.214, 1.483)	0.145
No	22 (84.615%)	4 (57.143%)		
Yes	4 (15.385%)	3 (42.857%)		
Internal fixation			0.677 (−0.174, 1.527)	0.607
Triple nail	12 (46.154%)	4 (57.143%)		
Double nail	3 (11.538%)	0 (0.000%)		
DHS	7 (26.923%)	1 (14.286%)		
FNS	4 (15.385%)	2 (28.571%)		
Serum calcium	7.841 ± 19.347	2.183 ± 0.233	0.414 (−0.427, 1.254)	0.108
creatinine	63.287 ± 21.622	66.957 ± 10.982	0.214 (−0.622, 1.050)	0.692
CRP	6.505 ± 8.723	7.861 ± 6.912	0.172 (−0.663, 1.008)	0.311
TAG	1.927 ± 0.916	2.689 ± 0.947	0.818 (−0.040, 1.676)	0.028
LDL-C	2.508 ± 0.954	2.484 ± 0.278	0.034 (−0.801, 0.869)	0.877
ALT	42.308 ± 34.510	27.429 ± 9.761	0.587 (−0.260, 1.433)	0.39
AST	27.077 ± 15.205	22.143 ± 4.100	0.443 (−0.398, 1.285)	0.774
D-D	0.370 ± 0.234	0.379 ± 0.097	0.050 (−0.788, 0.888)	0.114
RF	19.681 ± 5.412	21.843 ± 10.167	0.265 (−0.572, 1.103)	0.944
NSA	134.587 ± 9.193	119.407 ± 3.697	2.166 (1.182, 3.151)	<0.001
Hip preservation time (months)	46.423 ± 19.138	15.286 ± 8.499	2.103 (1.126, 3.080)	<0.001

The factors of gender, etiology, drinking history, smoking history, hormone use history, internal fixation method, and serum biochemical indicators (CRP, ALT, AST, etc.) did not show a significant impact on the outcome of hip preservation (p > 0.05). However, age and BMI were found to be significantly associated with the outcome of hip preservation (P < 0.05). Notably, the average NSA was 134.587° ± 9.193° in the survival group and 119.407° ± 3.697° in the failure group; thus indicating that NSA had a substantial influence on the outcome of hip preservation (P < 0.01). Consequently AGE,BMI,and NSA were subjected to further analysis.

**FIGURE 6:**
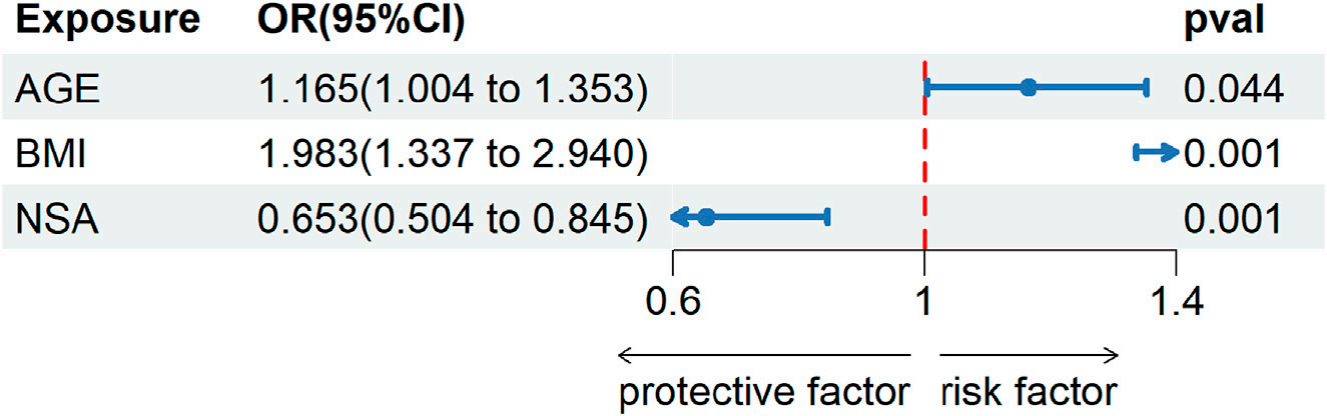
The figure displays the results of univariate logistic regression analysis, including the effects of age, BMI, and NSA on hip preservation outcomes. The horizontal axis represents the odds ratio (OR) and its 95% confidence interval, with a vertical line indicating an OR of 1. Each factor is represented by a point indicating the OR value, with the line segment showing the 95% confidence interval. As shown in the figure, age and BMI are identified as risk factors (OR > 1), whereas NSA is identified as a protective factor (OR < 1).

The forest map showed the results of univariate logistic regression analysis for AGE, BMI and NSA, where AGE [OR = 0.608, (95%CI, 0.093–3.985); P = 0.604] and BMI [OR = 0.458, (95%CI, 0.28–5.362); P = 0.458] was the risk factor for hip preservation outcomes, while NSA [OR = 0.608, (95% CI, 0.093–3.985); P = 0.604] was the protective factor for hip preservation outcomes (Figure 6).

Finite element analysis:According to the displacement cloud map of the femoral head in different NSA models after SHD-FNRO (transparent model represents before compression, solid model represents after compression displacement), the weight-bearing area of the femoral head exhibits the largest displacement which gradually decreases layer by layer. The maximum displacement after SHD-FNRO decreases with an increase in cervical trunk angle, however, it significantly increases when NSA >160°. This indicates that as the cervical trunk angle increases, the displacement of the femoral head following rotary osteotomy decreases and stability improves (Figure 6).

**FIGURE 7:**
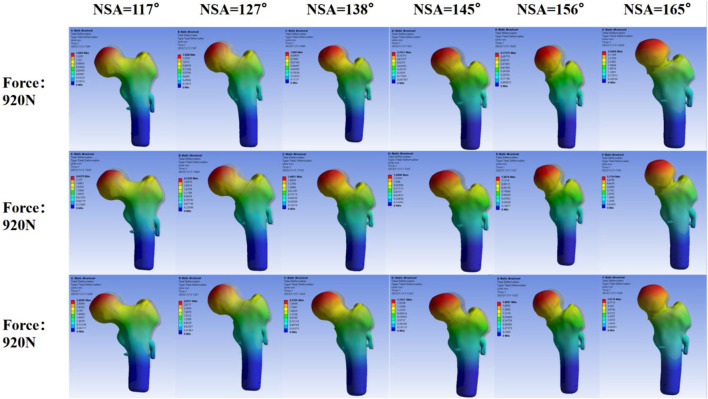
Maximum Displacement of the Femoral Head. This figure illustrates the maximum displacement of the femoral head under varying Neck-Shaft Angle (NSA) values (117°, 127°, 138°, 145°, 156°, and 165°). The displacement is represented by a color gradient, with blue indicating low displacement and red indicating high displacement. It is evident that as the NSA increases, the maximum displacement of the femoral head gradually decreases, and the stress distribution becomes more uniform. However, when the NSA exceeds 160°, the maximum displacement and stress concentration areas show a significant increase, suggesting potential instability in the postoperative outcomes.

**FIGURE 8:**
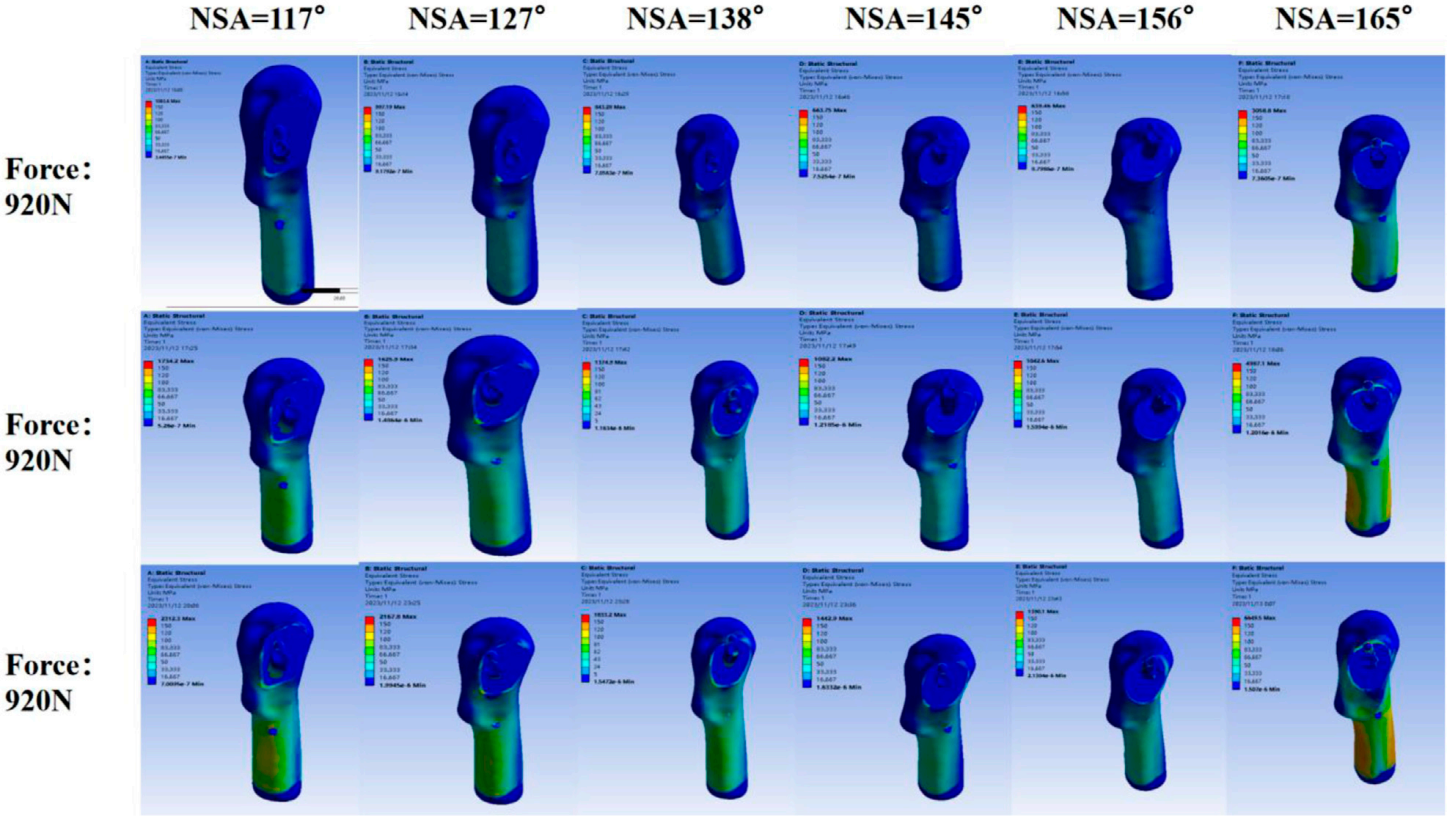
Stress Distribution on the Femoral Head. This figure depicts the stress distribution across the femoral head under different NSA values (117°, 127°, 138°, 145°, 156°, and 165°). The stress is illustrated using a color gradient, ranging from blue (low stress) to red (high stress). As the NSA increases, the stress distribution becomes more even. However, beyond an NSA of 160°, the stress concentration areas become more pronounced, indicating a higher risk of postoperative instability.

**FIGURE 9:**
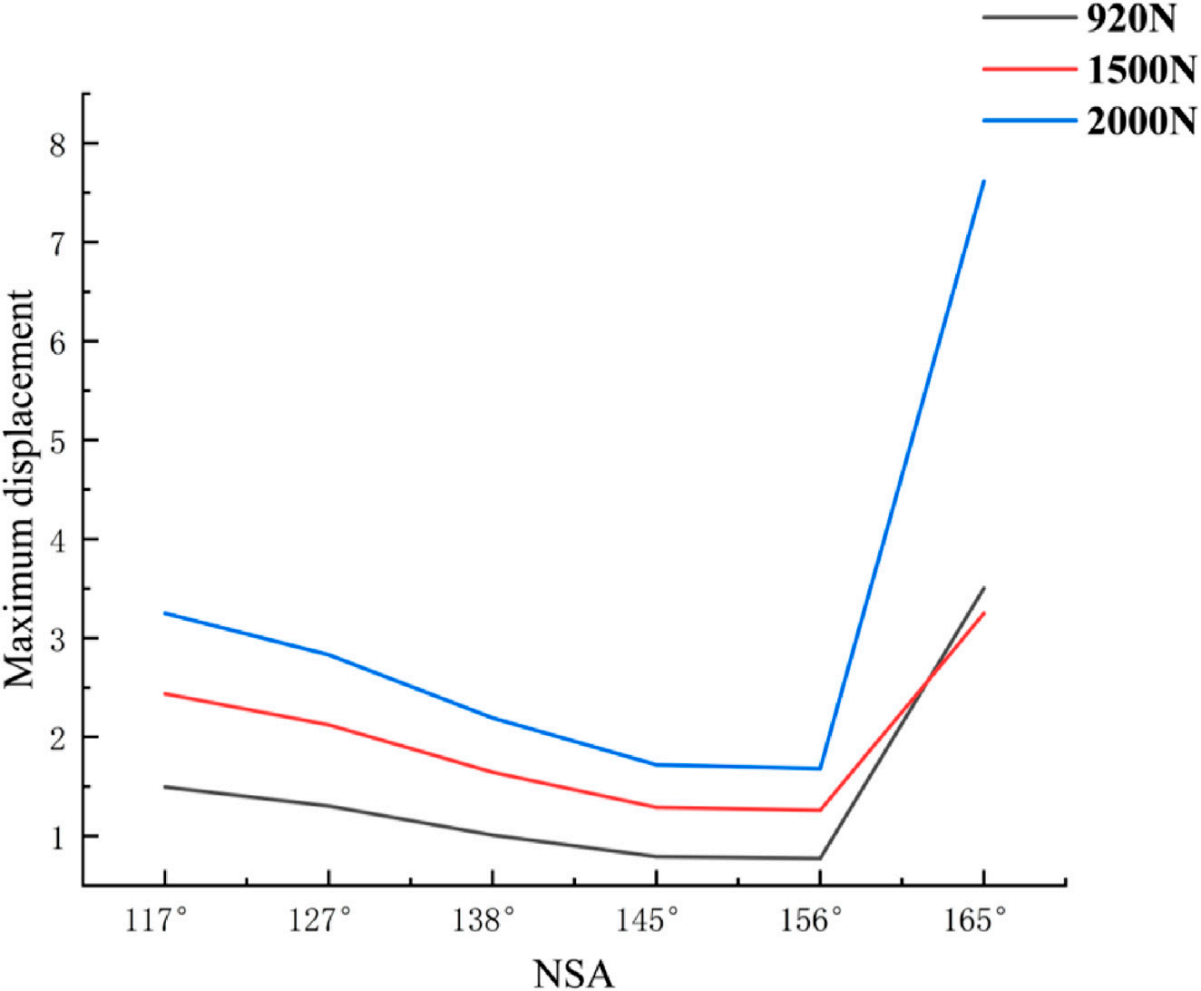
Postoperative Instability Due to Excessive NSA. This figure summarizes the effects of excessive NSA values on the femoral head.It demonstrates that while an optimal NSA range (between 117° and 160°) leads to reduced displacement and even stress distribution, an NSA exceeding 160° results in significant increases in both maximum displacement and stress concentration.

The stress nephogram of the femoral head after SHD-FNRO reveals a high-stress distribution at the junction between the femoral head and neck. The stress peak following SHD-FNRO appears on the osteotomy surface. Amongst all six models tested, when NSA = 117° is compared to others, higher stress concentration is observed at their junctions between head and neck along with an increase in overall stress concentration. The remaining five models exhibit multiple stress concentrations as depicted in Figures 7-9.

These findings suggest that different cervical trunk angles have significant effects on both displacement and stress distribution within the femoral head post-SHD-FNRO surgery. These factors should be thoroughly considered while selecting surgical methods and designing treatment plans to optimize surgical outcomes while minimizing potential risks.

## 4 Discussion

In this study, we conducted an analysis of the factors influencing postoperative outcomes. Statistical analysis of 33 patients who underwent rotational basal osteotomy for hip preservation revealed that gender, etiology, and serum biochemical indicators had no impact on the success rate of surgery. Our findings indicate that AGE, BMI, and NSA are significant factors affecting the outcome of hip preservation. As AGE and BMI increase, so does the difficulty and risk associated with hip preservation, which should be considered when deciding between hip preservation or replacement. These results align with previous studies (Erivan et al., 2020). However, Migliorini et al. (2023) reviewed data from 88 articles and found no statistically significant evidence linking patient age and BMI, etiology, time from surgery to full weight-bearing, or side to the results of hip preservation surgery. Therefore, further exploration is needed to understand the relationship between AGE/BMI and the outcome of hip preservation.

Additionally, our study revealed a close correlation between NSA size and postoperative efficacy as well as the success of hip preservation. Maintaining a normal or even larger range for NSA resulted in better outcomes. When NSA exceeded reasonable limits for successful hip preservation (either too large or too small), it led to poor postoperative efficacy or even failure. Similarly, Jiang et al. (2024) analyzed data from 1066 non-elderly patients with femoral neck fracture in the Chinese National Femoral Neck Fracture database through a multicenter retrospective study. They found that contralateral NSA below 130° was significantly associated with an increased risk of clinical failure (including nonunion, osteonecrosis of the femoral head, functional failure, and reoperation).

Based on the aforementioned studies, we conducted further investigations into the impact of different NSAs on the displacement and stress distribution of the femoral head following SHD-FNRO. Through finite element analysis, it was observed that smaller NSAs were prone to stress concentration on the osteotomy surface, which affected its healing and could potentially lead to loosening of internal fixation or cortical bone herniation in the femoral head. In our current study, one patient experienced screw dislocation and two patients had nonunion of the osteotomy surface, all with an NSA less than 120°. The size of NSA is closely associated with femoral head rotation and osteotomy angle. Therefore, surgeons should pay attention to selecting appropriate rotation angles during surgery while ensuring alignment of the osteotomy plane and considering NSA size. Finite element analysis results demonstrated a decrease in maximum displacement as NSA increased within 120°–160° range. In a mechanical analysis by Kainz et al. (2023) they established 25 musculoskeletal models with varying NSAs (93˚-153°), revealing that larger NSAs (153°) caused hip joint contact force to increase fivefold body weight, whereas lower NSAs (108° and 93°) resulted in a twofold increase in second peak knee joint contact force. We plan to conduct further evaluations involving a larger number of patients to validate this observation.

Additionally, we compared the material properties of necrotic and cancellous bone in the same model and found that the differences in maximum displacement and maximum stress of the femoral head were not significant (Supplementary Figure S1). We believe that the SHD-FNRO procedure, by rotating the osteotomy to transfer the necrotic area to a non-weight-bearing region (such as the posterior-medial aspect of the femoral head), significantly reduces its mechanical contribution. According to the principles of the Sugioka procedure, the necrotic area no longer bears the primary load postoperatively, and thus its material properties have a limited impact on the overall stress distribution of the model. However, actual necrotic areas may be accompanied by microstructural collapse or vascular regeneration, and future studies should incorporate micro-CT and multiscale modeling to further refine the analysis.

Nevertheless, this study has certain limitations. First, due to the technical difficulty of the SHD-FNRO procedure, the number of patients who underwent this surgery is small, and we lost a significant number of patients due to incomplete follow-up. Second, the positions and angles of the postoperative radiographs varied, leading to some errors in our data. Third, the material properties assigned to the femoral models were fixed, and we did not consider the influence of ligaments on femoral loading. Additionally, there is a lack of relevant biomechanical studies to fully validate our model. Fourth, this study focused on ARCO stage III patients, and the necrotic area was standardized to some extent through staging criteria. However, we did not deeply investigate the interaction between the necrotic area size and NSA. Future studies could explore the dynamic relationship between necrotic area size and NSA. Moreover, we only compared differences in mechanical factors among models with different NSAs and did not consider the effects of surgical approaches, blood supply, or osteotomy healing on postoperative outcomes. More mechanical factors and measures specific to the femoral head necrosis area should be considered. Finally, we will further investigate whether there are differences between the simulated postoperative model forces and the actual forces experienced by patients after surgery.

In conclusion, our findings suggest that NSA serves as an effective indicator for predicting hip preservation outcomes in AVascular necrosis patients. By assessing postoperative NSA perspectives accurately predict patient survival status and time which aids surgical planning efforts. Biomechanical results demonstrate that different NSA significantly impact displacement and stress distribution within the femoral head following SHD-FNRO.

## Data Availability

The original contributions presented in the study are included in the article/Supplementary Material, further inquiries can be directed to the corresponding authors.

## References

[B1] Brown T. D. Pedersen D. R. Baker K. J. Brand R. A. (1993). Mechanical consequences of core drilling and bone-grafting on osteonecrosis of the femoral head. J. Bone and Jt. Surg. Am. Volume 75 (9), 1358–1367. 10.2106/00004623-199309000-00011 8408157

[B2] Cardín-Pereda A. García-Sánchez D. Terán-Villagrá N. Alfonso-Fernández A. Fakkas M. Garcés-Zarzalejo C. (2022). Osteonecrosis of the femoral head: a multidisciplinary approach in diagnostic accuracy. Diagnostics 12 (7), 1731. 10.3390/diagnostics12071731 35885636 PMC9324583

[B3] Chau W. W. Ng J. P. Lau H. W. Ong M. T. Y. Chung K. Y. Ho K. K. W. (2021). Osteonecrosis of the hip: is there a difference in the survivorship of total hip arthroplasty with or without previous vascular iliac bone grafting? J. Orthop. Surg. Res. 16 (1), 244. 10.1186/s13018-021-02332-6 33832513 PMC8028070

[B4] Erivan R. Riouach H. Villatte G. Pereira B. Descamps S. Boisgard S. (2020). Hip preserving surgery for avascular hip necrosis: does terminating exposure to known risk factors improve survival? Phys. Sportsmed. 48 (3), 335–341. 10.1080/00913847.2020.1711827 31914339

[B5] Fang L. Qi J. Wang Z. Liu J. Zhao T. Lin Y. (2023). Inverse relationship between femoral lateralization and neck-shaft angle is a joint event after intramedullary nailing of per trochanteric fractures. Sci. Rep. 13 (1), 10999. 10.1038/s41598-023-38209-3 37419961 PMC10328961

[B6] Jiang D. Zhu H. Cao J. Cai Q. Wu F. Li X. (2024). Contralateral neck-shaft angle lower than 130° is associated with clinical failure in nongeriatric individuals: analysis of the national femoral neck fracture database of 1066 patients. Clin. Orthop. Relat. Res. 482 (10), 1801–1812. 10.1097/CORR.0000000000003071 38662919 PMC11419447

[B7] Kainz H. Mindler G. T. Kranzl A. (2023). Influence of femoral anteversion angle and neck-shaft angle on muscle forces and joint loading during walking. PLoS One 18 (10), e0291458. 10.1371/journal.pone.0291458 37824447 PMC10569567

[B8] Migliorini F. Maffulli N. Baroncini A. Eschweiler J. Tingart M. Betsch M. (2023). Prognostic factors in the management of osteonecrosis of the femoral head: a systematic review. Surgeon 21 (2), 85–98. 10.1016/j.surge.2021.12.004 34991986

[B9] Oh Y. Fujita K. Wakabayashi Y. Kurosa Y. Okawa A. (2017). Location of atypical femoral fracture can be determined by tensile stress distribution influenced by femoral bowing and neck-shaft angle: a CT-based nonlinear finite element analysis model for the assessment of femoral shaft loading stress. Injury 48 (12), 2736–2743. 10.1016/j.injury.2017.09.023 28982480

[B10] Song H. Zhang D. Wang P. (2023). Finite element analysis of rotational osteotomy of the base of femoral neck fixed by femoral neck internal fixation system. Bioorthopaedic Mater. and Clin. Res. 20 (03), 12–18.

[B11] Sugioka Y. Hotokebuchi T. Tsutsui H. (1992). Transtrochanteric anterior rotational osteotomy for idiopathic and steroid-induced necrosis of the femoral head: indications and long-term results. Clin. Orthop. Relat. Res., 277. 1555330

[B12] Xue Z. Sun J. Li T. Huang Z. Chen W. (2019). How to evaluate the clinical outcome of joint-preserving treatment for osteonecrosis of the femoral head: development of a core outcome set. J. Orthop. Surg. Res. 14 (1), 317. 10.1186/s13018-019-1364-x 31597557 PMC6785903

[B13] Yadav R. N. Oravec D. J. Morrison C. K. Bevins N. B. Rao S. D. Yeni Y. N. (2023). Digital wrist tomosynthesis (DWT)-based finite element analysis of ultra-distal radius differentiates patients with and without a history of osteoporotic fracture. Bone 177, 116901. 10.1016/j.bone.2023.116901 37714502

