# Supplementary figures and images for: To investigate the effect of neck-shaft angle in surgical hip dislocation combined with femoral neck rotational osteotomy in the treatment of osteonecrosis of the femoral head and to combine with finite element analysis
Source: Front Bioeng Biotechnol. 2025 Mar 19;13:1495292. doi: 10.3389/fbioe.2025.1495292 (PMC11962004; doi:10.3389/fbioe.2025.1495292)

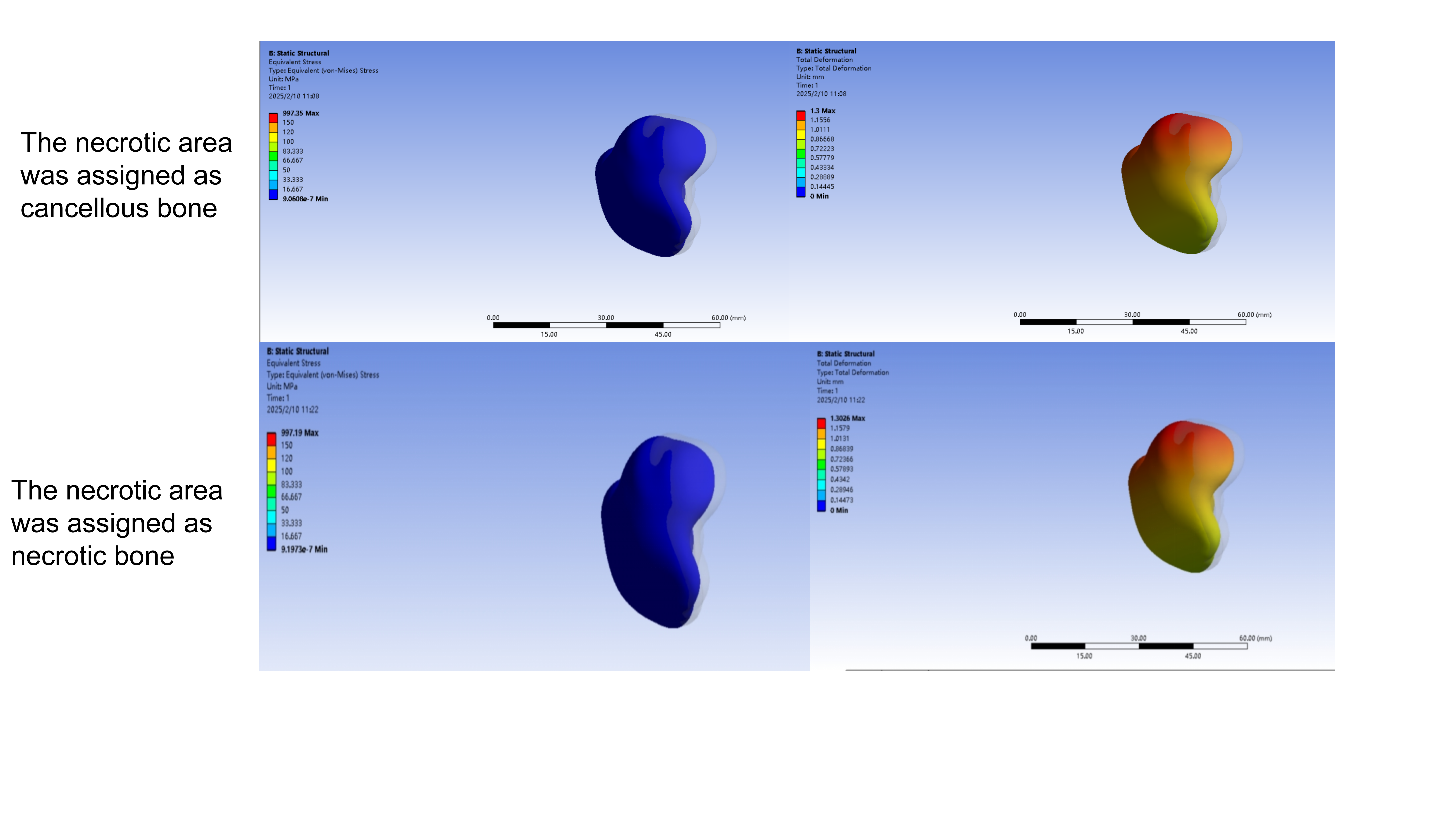

Supplement: Supplementary file 1 [file Image1.png]
